# Inhibiting mtDNA‐STING‐NLRP3/IL‐1β axis‐mediated neutrophil infiltration protects neurons in Alzheimer's disease

**DOI:** 10.1111/cpr.13529

**Published:** 2023-08-01

**Authors:** Xiangyu Xia, Xuemei He, Tingmei Zhao, Jingyun Yang, Zhenfei Bi, Qianmei Fu, Jian Liu, Danyi Ao, Yuquan Wei, Xiawei Wei

**Affiliations:** ^1^ Laboratory of Aging Research and Cancer Drug Target, State Key Laboratory of Biotherapy and Cancer Center, National Clinical Research Center for Geriatrics, West China Hospital Sichuan University Chengdu Sichuan China

## Abstract

Neutrophil is a pathophysiological character in Alzheimer's disease. The pathogen for neutrophil activation in cerebral tissue is the accumulated amyloid protein. In our present study, neutrophils infiltrate into the cerebra in two models (transgenic model APP/PS1 and stereotactic injection model) and promote neuron apoptosis, releasing their cellular constituents, including mitochondria and mitochondrial DNA (mtDNA). We found that both Aβ_1–42_ and mtDNA could provoke neutrophil infiltration into the cerebra, and they had synergistic effects when they presented together. This neutrophillic neuroinflammation upregulates expressions of STING, NLRP3 and IL‐1β. These inflammatory cytokines with mtDNA constitute the mtDNA‐STING‐NLRP3/IL‐1β axis, which is the prerequisite for neutrophil infiltration. When any factor in this pathway is depleted, the migration of neutrophils into cerebral tissue is ceased, with neurons and cognitive function being protected. Thus, we provide a novel perspective to alleviate the progression of Alzheimer's disease.

## Graphical abstract

The schematic pathogenesis of Aβ induced neutrophil neuroinflammation is as follows. Since Aβ interacted with neurons, cellular apoptosis and necrosis initiated. And then mitochondrial DNA (mtDNA) is released from these impaired cells. After being captured by neutrophils, free mtDNA binds to STING protein and activates it. The activated STING protein induces increased expressions of downstream NLRP3 and IL‐1β. With released mtDNA, NLRP3 and IL‐1β captured by more neutrophils, numerous neutrophils infiltrate into Aβ accumulated regionand aggravate neuroinflammation. This cycle is repeated continuously, which eventually induces neutrophil neuroinflammation and damages neural tissue.

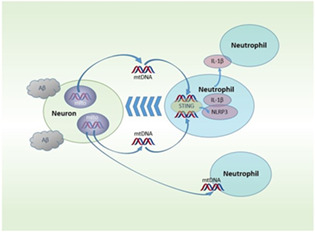

## 1 INTRODUCTION

Alzheimer's Disease was primarily reported by Alois in 1907.^1^ It is characterized by cognitive impairment and dementia in patients.^2^, ^3^, ^4^ Pathologically, it is defined as neuron damage, amyloid protein accumulation and neuroinflammation activation in the central nervous system.^5^, ^6^, ^7^, ^8^ Early research suggested that the damage to the central nervous system in patients was mainly caused by the accumulation of amyloid protein, which was essentially constituted by Aβ (Amyloid beta) and Tau.^9^, ^10^, ^11^ Aβ derived from abnormal hydrolysis of APP (Amyloid beta Precursor Protein) protein. APP was hydrolyzed and regulated by α‐secretase and γ‐secretase in physiological conditions. But in Alzheimer's disease, APP was hydrolyzed by β‐secretase and γ‐secretase, which produced Aβ.^5^ Aβ had toxicity to neuron cells in various ways, interfering with cellular metabolism and impairing their physiological functions.^6^, ^7^, ^12^, ^13^, ^14^ Recently, a phase‐3 trial with 1795 patients demonstrated that Lecanemab, a humanized monoclonal antibody bound to and eliminated soluble Aβ, could reduce clinical decline in global cognition by 27% compared with the placebo group.^15^ When neuron cells are damaged by amyloid proteins, their contents were released and neuroinflammation was activated. In the past 10 years, researchers revealed that neuroinflammation was also an important factor causing damage to the central nervous system, and the damage caused by it was even more severe than that caused by amyloid protein.^5^, ^8^, ^16^, ^17^, ^18^

The major cell responsible for neuroinflammation was described as microglia.^19^, ^20^, ^21^ Recent studies found that neutrophils are also involved in the pathogenesis of Alzheimer's disease.^22^, ^23^, ^24^, ^25^ Alzheimer's model animals upregulated the expressions of vascular endothelial adhesion factors such as VCAM‐1, ICAM‐1 and LFA‐1, which induced neutrophil infiltration and impaired cognition. When neutrophils were deprived, cerebral tissue was protected and cognitive function was improved.^22^

In addition, when neuron cells disintegrated, numerous mitochondrial DNA (mtDNA) was released. mtDNA was evolutionarily homologous to bacteria DNA. Thus, it could activate the immune system.^26^, ^27^, ^28^, ^29^, ^30^ In our previous studies, we found that mitochondria and mtDNA were important factors for inflammatory responses, with the capability to attract neutrophils to the lesion and exacerbate damage.^26^ In this research, we found that Aβ could induce impaired neurons activating the mtDNA‐STING‐NLRP3/IL‐1β axis, initiate neutrophil infiltration in cerebra and induce central nervous system damage. With the knockout of relevant genes on this axis, the cerebral tissue was protected. Thereby, we revealed that in the pathogenesis course of Alzheimer's disease, amyloid protein acted as the initiator, and the activation of mtDNA‐STING‐NLRP3/IL‐1β axis could induce subsequent damage to the nervous system.

## 2 MATERIALS AND METHODS

### 2.1 Mice and cell lines

C57BL/6 mice aged 6–8 weeks were obtained from Charles River. *Sting* ^−/−^ knockout mice were purchased from The Jackson Laboratory, *IL‐1β* ^−/−^ and *Nlrp3* ^−/−^ knockout mice were provided by the University of Tokyo. The 8‐month APP/PS1 double transgenic mice were purchased from Sairuike Biotechnology Co., Ltd (Wuhan), which had been identified as co‐overexpressing the *APP* and *PS1* genes. All animals were fed in specific pathogen‐free (SPF) animal house and used strictly according to the treaty of the Sichuan University Animal Protection and Ethics Association.

Mice hippocampal neuron cell line HT22 was obtained from Procell Life Technology Co., Ltd (Wuhan). N2a and SK‐N‐BE cell lines were from the cell bank of Sichuan University.

### 2.2 Mice tissue preparation and immunofluorescent assay

Mice were sacrificed for tissue harvest. Cut the sternum and ribs of the mice to expose the heart and perfused with ice‐cold normal saline from the left ventricle. The collected samples were stored at −80°C until required for section or other experiments. Embedded mice brains with OCT and freeze at −20°C. Then sliced brain tissue with a freezing slicer with a thickness of 40 μm (LEICA, Germany). The frozen tissue sections were dried in the air for the staining process or stored at −20°C. Washed sections with PBS (phosphate buffered solution) for 5 min. 1% Triton‐100 drilled for 15 min. PBS washed three times, 5 min each time. 5% BSA (bovine serum albumin) blocked for 30 min. PBS washed three times, 5 min each time. Added primary antibody to cover the tissues (anti‐Aβ antibody, 1:1000, Abcam, USA; anti‐Ly6G antibody, 1:2000, Abcam, USA; anti‐DNA/RNA damage 1:1000, Abcam, USA) at 4°C overnight. PBS washing three times, 5 min each time. Dripped fluorescent secondary antibodies (anti‐rabbit FITC antibody, Abcam, USA; anti‐rat TRITC antibody, Abcam, USA; anti‐mouse FITC/TRITC antibody, Abcam, USA) onto the sections until covered completely, then incubated them in room temperature for 2 h. Used PBS to wash secondary antibodies and dripped DAPI. If primary antibodies are the same host species for a single slice, the procedure is to drip one sort of primary antibody and washed for one colour of the secondary antibody and washed this secondary antibody for another primary antibody and a different colour of secondary antibody. The experiment in vitro used mice hippocampal neurons HT22 cells, cultured with DMEM medium (Gibco, USA) on the 24‐well climbing sheet (WHB scientific, China). After 24 h of administration, cells were fixed with polyformaldehyde solution. After that, cells were washed with PBS for 5 min. PBS solution with 1% Triton‐100 drilled for 15 min. PBS washed three times. 5% BSA sealed for 30 min. PBS washed three times. Diluted the primary antibody (anti‐Caspase‐3 antibody 1:1000, CST, USA) and dripped it onto a climbing sheet covering it completely and stayed at 4°C overnight. PBS washed three times and dripped fluorescent secondary (anti‐rabbit FITC antibody, Abcam, USA) antibody and incubated at room temperature for 2 h. Used PBS to wash secondary antibodies and dripped DAPI. Then took photographs with the microscope (DM6B, LEICA, Germany) and randomly select five high power fields to count the total number of positive cells for statistics.

### 2.3 Stereotactic injection

Mice were transferred into the isoflurane (RWD life technology. Ltd, China) anaesthesia box until they were completely anaesthetized. Fixed the upper incisor and external auditory meatus of the mice and maintained them in sedation with isoflurane on the stereotaxic instrument (RWD life technology. Ltd, China). Sterilized the head skin of mice twice with 75% medical alcohol and cut the skin. Then, located the position of the cerebral cortex and hippocampus according to the following bregma coordinates: the cerebral cortex: −2.2 mm, anteroposterior; 1.7 mm, mediolateral; 0.8 mm, dorsoventral; CA1: −2.2 mm, anteroposterior; 1.7 mm, mediolateral; and 1.5 mm, dorsoventral; DG (dentate gyrus): −2.2 mm, anteroposterior; 1.7 mm, mediolateral; and 2.2 mm, dorsoventral. Drilled the mice skull with drill (78,001, RWD life technology. Ltd, China) and injected a total volume of 2 μL of liquid into the cortex (1 μL), CA1 (0.5 μL) and DG (0.5 μL) regions at one hemisphere, 0.5 μL /min. Once the one side of the hemisphere injection was completed, injecting the other side. Cells: 10^6^/ml, fMLF: 1 μmol/L (Sigma, USA), Aβ_1–42_: 0.44 mg/mL, mitochondria: 50 mg/mL, mitochondrial DNA: 1 mg/mL, mixture (Aβ_1–42_ and mtDNA): Aβ_1–42_: 0.44 mg/mL and mtDNA 1 mg/mL. After the injection, slowly pulled out the needle, sutured the scalp and sterilized twice. Put mice back into the cage until they recovered.

### 2.4 Aβ_1–42_ preparation

Aβ_1–42_ (QYAOBIO, China) was dissolved in hexafluoroisopropanol (Sigma, USA), and gently shaken until no visible solid, then stored at 4°C overnight. Dried hexafluoroisopropanol in the fume hood and added dimethyl sulfoxide (DMSO) into Aβ_1–42_ containers to dissolve it. Vortex Aβ_1–42_ solution until there was no visible precipitation. Incubated the solution at 4°C overnight. Centrifuged the solution at 12000 rpm, 4°C for 10 min. The supernatant was soluble Aβ_1–42_ and transferred the supernatant into a clean container. Used the BCA assay kit (ThermoFisher Scientific, USA) for concentration detection, then stored the sample at 4°C.

### 2.5 Preparation of mtDNA

Collected 2–5 g of fresh mice brain tissue and washed in TE (Tris‐EDTA) buffer twice. Ground mice brain into homogenate with a grinder (Servicebio, China). Centrifuged the homogenate at 1000 rpm for 10 min, collected supernatant and repeated this step three times. The supernatant was centrifuged at 12000 rpm for 10 min, and the precipitate was crude mitochondria, washed the sediment twice with TE buffer. Prepare mitochondrial DNA lysate solution (NaOH 0.2 mol/L; 1% SDS v/w). Divided the crude mitochondria into 10 parts, added about 1 mL of lysate solution into each part and resuspended. Heated the solution at 65°C for 24 h. Then added the same volume of saturated phenol into the lysate solution, mixed them well, placed on the oscillator for 15 min, added chloroform with the same volume of phenol into the mixture and shaken for 15 min. Centrifuged solution at 12000 rpm for 5 min, collected the upper water phase solution and transferred them into clean containers. Added twofold the volume of anhydrous ethanol to the solution at 4°C, 30 min. Centrifuged the solution at 12000 rpm 4°C for 30 min, and the white flocculent precipitate was mitochondrial DNA. Washed mitochondrial DNA twice with anhydrous ethanol and dissolved with TE buffer. Confirmed the concentration of mtDNA (Nanodrop 2000, Thermo, USA) and store at 4°C.

### 2.6 Preparation for damaged cells and their mitochondria, mtDNA。

HT22 cells were cultured to the logarithmic growth phase. Added 8 μmol/L Aβ_1–42_ and incubated for 24 h. Collected cells and supernatant. The supernatant was centrifuged at 12000 rpm for 30 min to collect mitochondria leaked from damaged cells. Part of mitochondria was used to extract mtDNA with the methods described above. The Aβ_1–42_‐treated HT22 cells and their mitochondria, mtDNA were stored at −20°C for use.

### 2.7 Flow cytometry

This experiment mainly used the apoptosis detection kit (BD Biosciences, USA), the brief steps were as follows: HT22 cells were cultured with DMEM medium on 24‐well plates, when cells stayed in the logarithmic growth phase and added Aβ_1–42_ into cultures. After 24 h, collecting cells and supernatant were in each well and centrifuged at 1000 rpm for 5 min. Discarded the supernatant and used PBS resuspended cells and centrifuged at 1000 rpm for 5 min. Discarded the supernatant and added 300 μL binding buffer to resuspended cells. Added 5 μL PI and AnnexinV‐FITC into each tube and incubated in the dark at room temperature for 15 min. Centrifuged at 1000 rpm for 5 min, discarded the supernatant and used PBS resuspended cells. After the last centrifugation, add 100 μL 1× Binding Buffer for detection by flow cytometer (ACEA Biosciences, Inc., USA).

### 2.8 Identification of mitochondrial DNA with quantitative PCR

HT22, N2a and SK‐N‐BE cells were cultured with DMEM. When they were in the logarithmic growth phase, added Aβ_1–42_ into the culture. Incubated for 24 h and collected supernatant. We used synthesized sense primer sequence as 5 ‘‐ GCCCAGATAGCATTCC‐3’, antisense primer as 5 ‘‐ GTTCATCCTGTTCCTGCTCC‐3’, fluorescent probe as 5’‐ATGAGTTCCCCTACCAATACCACACCC‐3′. Added sample 7 μL, sense primer 1 μL, antisense primer 1 μL, probe 1 μL and TaqDNA Polymerase 10 μL into the reaction tubes. Used the following parameters to amplify mtDNA with real‐time qPCR (BIO‐RAD, USA): 95°C 120 s for 1 cycle, 95°C 5 s for 39 cycle, 55°C 10 s for 39 cycle, 65°C 5 s for 1 cycle, 95°C 120 s for 1 cycle. Then cooled to room temperature and process the data.

### 2.9 ELISA for cytokines

This experiment used the ELISA (enzyme‐linked immunosorbent assay) kit (ThermoFisher Scientific, USA), and brief protocols were as follows: added 100 μL diluted capture antibody solution into each clean 96‐well plate well, overnight at 4°C. Discarded the solution in each well, washed by PBST (phosphate buffered solution with 0.1% Tween20) for three times. Added 100 μL of ELISA/ELISPOT diluent to each well to reduce non‐specific reaction. Incubated at room temperature for 1 h. Discarded the solution and washed them by PBST three times. Added the standard and samples at 4°C overnight. Discarded the liquid and washed it by PBST three times. Added 100 μL primary antibody diluent into each well. Then sealed the plate and incubated at room temperature for 1 h. Discarded the liquid and washed it three times by PBST. Then, added 100 μL HRP‐coupled secondary antibody into each well and incubated at room temperature for 30 min. Discarded the liquid and washed it with PBST six times, then added 100 μL TMB solution and incubated at room temperature for 15 min. Added 100 μL reaction termination solution into each hole. Read the absorbance value of each well and draw the standard concentration curve. Then analyse the data.

### 2.10 Immunoblot

Collected the cells and tissues, then added RIPA (Beyotime, China) to cleavage cells and tissues. After that, centrifuged the samples at 12000 rpm, 4°C, 5 min. Collected the supernatant and used a BCA kit (ThermoFisher Scientific, USA) to detect the total concentration of protein. Then mixed the sample with a loading buffer at 95°C 10 min. Then stored them at −20°C. Prepared 12.5% SDS‐PAGE gel. Loaded sample protein 20–50 μg each well and then conducted electrophoresis at 100 V voltage, and stopped electrophoresis when the indicator reached the bottom. Cut the PVDF film (Merck Millipore, USA) to the proper size and soaked it in methanol for 10 s to activate it. Taken out the SDS‐PAGE gel for film‐transfer process at 200 mA for 1 h. After transferring completed, washed the PVDF film with TBST (tris‐buffered saline with 0.1% Tween20) and then put it into a 5% skimmed milk solution for sealing. Then, transferred PVDF film into the primary antibody solution (anti‐NLRP3 antibody, 1:2000, LSbio, USA; anti‐STING antibody, 1:2000, CST, USA; anti‐GAPDH and anti‐β‐actin antibody, 1:2000, Santa Cruz biotechnology, USA) at 4°C overnight. After that washed the PVDF film with TBST three times. Then immersed film with HRP coupled secondary antibody (anti‐rabbit IgG and anti‐mouse IgG, 1:10000, Invitrogen, USA) and incubated at room temperature for 2 h. After the secondary antibody incubation, dripped the luminescent liquid (ECL substrate, BIO‐RAD, USA) onto PVDF film, and conducted image collection process by bio‐rad imager (ChemiDoc, BIO‐RAD, USA).

### 2.11 TUNEL

This experiment used the commercial TUNEL (TdT‐mediated dUTP Nick‐End Labeling) kit (Promega, USA) and operated according to its instructions. The brief steps were as follows: fixed sections in 4% polyformaldehyde PBS for 15 min. Immersed sections in PBS twice for 5 min each time. Added 100 μL balance buffer at room temperature for 5–10 min. Dripped 50 μL TdT reaction mixture covering tissues. Immersed the sample in SSC solution to stop reaction for 15 min. Washed the sample in PBS three times. Sections were re‐stained with DAPI and observed by fluorescent microscope.

### 2.12 Image Quantification

Cerebral hippocampal sections of mice were sectioned at 40 μL thickness as described above. The analysis was performed by the image threshold adjust function of Image J 1.53e software (National Institutes of Health, USA). The brain regions of interest (Bregma −1.6 mm to −3.5 mm) with six sections per mouse were analysed. Nissl‐stained (Beyotime, China) sections were photographed by microscope system (Axiolab 5, Zeiss, Germany).

### 2.13 Neutrophil preparation and transwell assay

Collected mice neutrophils from their bone marrows. Isolated bone marrows from femur and tibia with sterile normal saline solution and gently repeatedly blew until there were no visible particles in the solution. Took a sterile 15 mL centrifuge tube, added Sigma cell separation solution (Sigma, USA) to the bottom and then gently dripped bone marrow solution onto it. Centrifuge at 800 g for 20 min to isolate neutrophils. More than 90% of the isolated cells were neutrophils as determined by flow cytometry. Washed neutrophils with RPMI‐1640 medium (Gibco, USA) once and resuspended them in RPMI‐1640 medium. Added the neutrophils (1 × 10^6^/ml) evenly into the inner chamber of the transwell plate (Corning, USA), and added an appropriate amount of RPMI‐1640 medium with Aβ_1–42_, mtDNA, and mixture of Aβ_1–42_/mtDNA into the outer chamber of the plate. After 24 h of incubation, collected the medium of the outer chambers, stained with haematoxylin and counted a number of neutrophil that migrated into the outer chamber.

### 2.14 Barnes maze test

The Barnes maze used a round disc that is 91 cm in diameter and 90 cm above the floor (SA208, Jiangsu Science Biological Technology Co. Ltd.). All mice from the fourth day before the test were placed in the starting box in the centre of the maze for 3 min, and then the mice were gently nudged into the target hole with the shelter box for 3 min. Then, from the third day before the test, the mice were placed in the central starting box of the maze every day and then released. If the mice could not find the shelter within 90 s, gently nudged mice into it and stayed for 3 min. From the first day of the test, the mice were placed in the central starting box of the maze, and the starting box was placed in the centre of the maze, then the strong light and noise stimulation were turned on. Then, mice were released, and the latency of mice successfully getting into the shelter box was recorded (latency, the longest stimulation time was 90 s). Each mouse was tested three times a day for three consecutive days.

### 2.15 Statistics

We used GraphPad Prism 8 to conduct statistical analysis of the data and created statistical charts. The results were expressed in mean ± standard error (SEM). The significant difference between samples was analysed by a *t* test method.

## 3 RESULTS

### 3.1 Aβ induces neutrophil infiltration and neuron impairment

**FIGURE 1:**
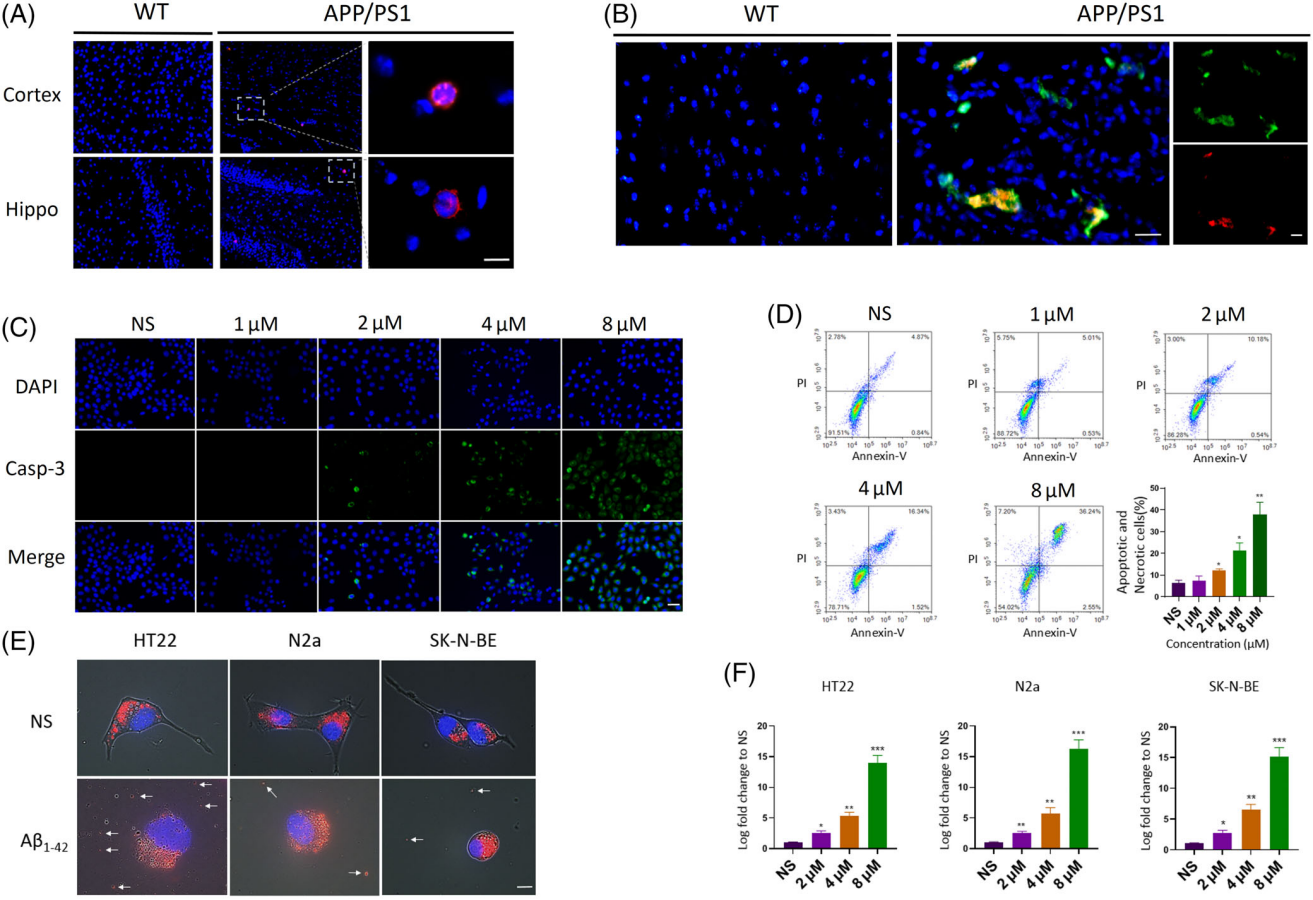
Aβ induced neutrophil infiltration and neuron impairment (A) Immunofluorescent images of Ly6G+ leukocytes (red cell, DAPI blue) in cerebral tissue with age‐matched 8‐month wild‐type (WT) control mice and APP/PS1 mice. Scale bar 10 μm. (B) The complex of Aβ (Green) and damaged DNA (Red) in 8‐month wild‐type and APP/PS1 mice. DAPI blue. Scale bar 25 μm. (C) The caspase‐3+ HT22 cells (Green cells) were present after 24 h treated with different concentrations of Aβ_1–42._ DAPI blue. Scale bar 75 μm. (D) The percentage of apoptotic and neurotic HT22 cells detected by flow cytometry after 24 h treated with different concentrations of Aβ_1–42_ and the statistic results. *n* = 3 per group. *0.01 < *p* < 0.05 versus NS; **0.001 < *p* < 0.01 versus NS; *t* test; data are means ± SEM. (E) Mitochondria leakage (stained by mito‐tracker, Red) from neuron cells or neuroblastoma cells after 24 h treated with 2 μmol/L Aβ_1–42_. Hoechst blue. Scale bar 10 μm. (F) The quantity of leaked mitochondria DNA from different cells detected by quantitative PCR. Cells were treated by normal saline, 2 μmol/L, 4 μmol/L and 8 μmol/L Aβ_1–42._ *n* = 3 per group. *0.01 < *p* < 0.05 versus NS; **0.001 < *p* < 0.01 versus NS; ***0.0001 < *p* < 0.001 versus NS; *t* test; data are means ± SEM.

We performed a genetically modified animal experiment to test the role of neutrophils in Aβ presence condition. APP/PS1 mice were characterized by highly expressing Aβ. We adopted 8 month APP/PS1 mice as observation subject, which had heavy accumulation in cerebra and were controlled by the wild‐type (WT) mice of the same age. Ly6G is the characteristic marker of neutrophil. The results revealed that Ly6 G‐positive cells were found in both cortex and hippocampus in APP/PS1 mice (Figure 1A). However, there was no significant infiltration of neutrophils in cerebra of WT mice, suggesting that neutrophils migration depended on the Aβ accumulation.

Next, we observed that a large amount of oxidative damaged DNA was presented in the cerebra of APP/PS1 mice. This damaged DNA was in the same place as the accumulated amyloid proteins (Figure 1B), suggesting that damaged DNA bound to Aβ to form the DNA‐Aβ complex.

To confirm Aβ impaired neuron cell will release intracellular DNA, we conducted in vitro experiments. First, we used mouse hippocampal neurons HT22 cell as the experimental subject and added different concentrations of Aβ_1–42_. At lower concentrations, the toxicity of Aβ_1–42_ was weak. With the increase in the concentration, the toxicity gradually enhanced. Besides, 2 μmol/L of Aβ_1–42_ could induce significantly increased expression of caspase‐3 in HT22 cells, which was a crucial marker for cellular apoptosis and necrosis (Figure 1C). Flow cytometry experiments had similar results in immunofluorescence (Figure 1D). All these results indicated that 2 μmol/L Aβ_1–42_ was the threshold concentration to induce toxicity for cells in vitro. Second, we took this margin concentration as a condition to observe the release of mitochondria directly. To exclude accidental phenomena, we conducted the experiment by three cell lines. We found that all cell lines (HT22, N2a and SK‐N‐BE) released mitochondria when exposed to Aβ_1–42_ (Figure 1E). Additionally, the concentrations of mitochondrial DNA (mtDNA) were increased with the higher concentration of Aβ_1–42_ (Figure 1F). Thus, Aβ_1–42_ could induce neutrophil infiltration, neuron apoptosis and necrosis. These impaired neurons released mitochondria and mtDNA. mtDNA could bind to amyloid proteins.

### 3.2 Neutrophil neuroinflammation activates inflammatory cytokines expression

**FIGURE 2:**
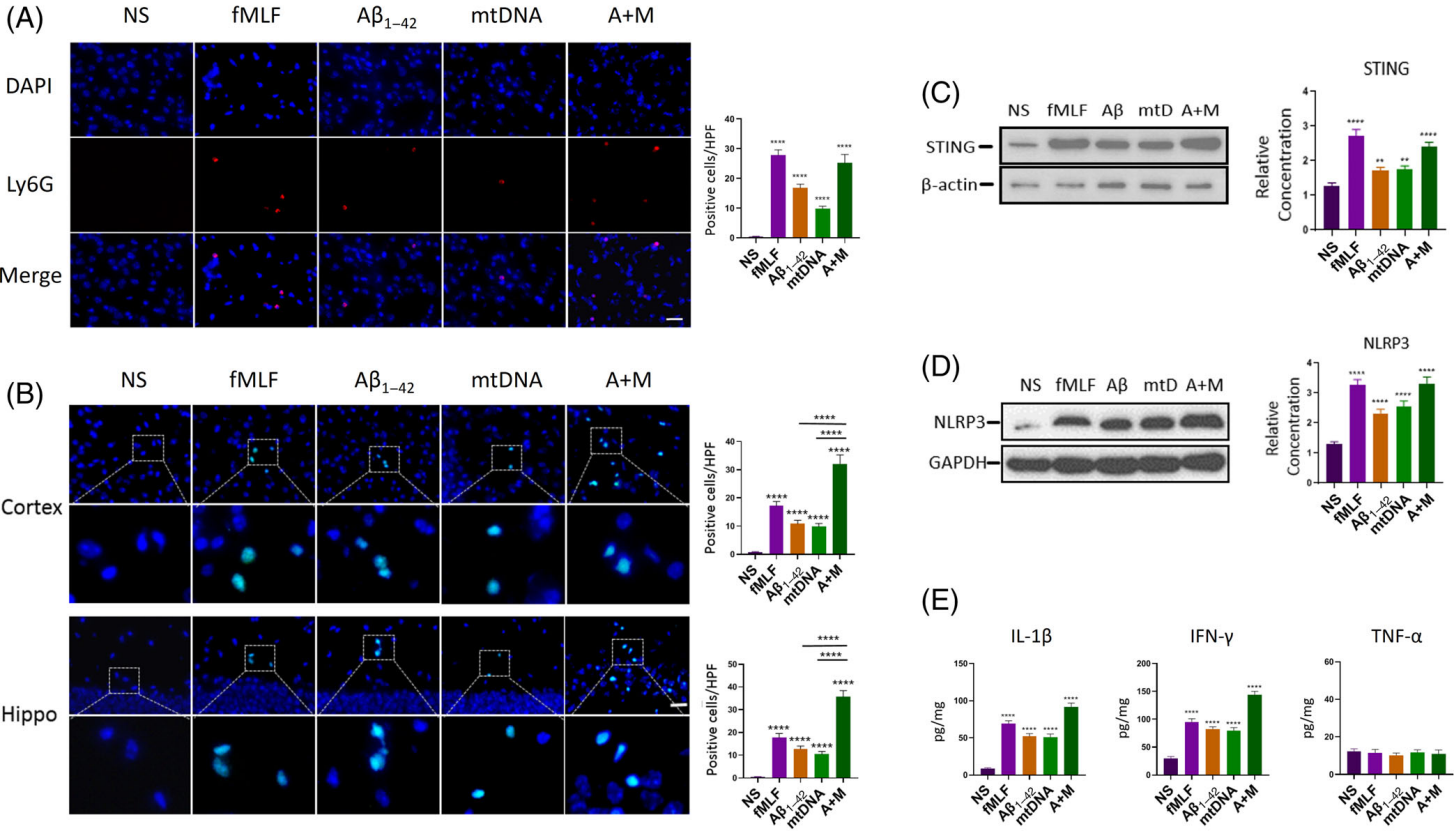
Inflammatory cytokines expression activated by Aβ_1–42_ and mtDNA‐induced neuroinflammation. (A) Neutrophil infiltrations in cerebral tissues in wild‐type mice after 24 h injection by NS, fMLF, Aβ_1–42_, mitochondrial DNA (mtDNA), the mixture of Aβ_1–42_ and mtDNA (A+M). *n* = 6 per group. ****p < 0.0001 versus NS; *t* test; data are means ± SEM. Scale bar 25 μm. (B) TUNEL+ cells (TdT‐mediated dUTP Nick‐End Labeling) in cortex and hippocampus with statistics after injection by 24 h. *n* = 6 per group. ****p < 0.0001 versus NS; *t* test; data are means ± SEM. Scale bar 25 μm. (C,D) STING and NLRP3 expressions in cerebral tissue detected by immunoblot assay and the grayscale relative concentration analysis. *n* = 6 per group. **0.001 < *p* < 0.01 versus NS; ****p < 0.0001 versus NS; *t* test; data are means ± SEM. (E) The concentration of IL‐1β, IFN‐γ and TNF‐α detected by ELISA (enzyme‐linked immunosorbent assay). *n* = 6 per group. ****p < 0.0001 versus NS; *t* test; data are means ± SEM.

Next, we investigated the toxic effect of Aβ_1–42_ on cerebra in vivo. We injected fMLF, Aβ_1–42_, mtDNA and Aβ/mtDNA mixture into the cerebral tissue of wild‐type C57BL/6 mice respectively. fMLF is a short peptide chain that is the chemotactic signal for neutrophil migration. In this experiment, fMLF was used as the positive control. We injected these subjects into the brain. We found that all these subjects could induce neutrophil infiltration (Figure 2A). Wherein, the number of neutrophils migrated by Aβ and mtDNA was basically even, while the number of neutrophils in Aβ/mtDNA mixture group was significantly higher than that in Aβ or mtDNA groups. Namely, Aβ and mtDNA could activate the migration of neutrophils into cerebral tissue. Besides they had synergistic effects in chemotactic function when they presented together.

To explore the toxicity of Aβ in vivo, we conducted TdT‐mediated dUTP Nick‐End Labeling (TUNEL) assay. When neurons apoptosis began, DNA in cells would be fragmented. The fragmented DNA could be detected by TdT‐mediated dUTP. We observed that the injections of fMLF, Aβ_1–42_, mtDNA and Aβ/mtDNA mixture could induce apoptosis (Figure 2B). Similarly to the results of Ly6G cells infiltration, the number of TUNEL‐positive cells in the Aβ/mtDNA mixture group was much higher than that in Aβ or mtDNA group. Interestingly, there was no evidence showing that fMLF had direct cellular toxicity to neurons, but it could induce a large number of neurons for apoptosis, suggesting that the toxicity of fMLF might come from the infiltrated neutrophils.

Furthermore, we noticed the homology between mtDNA and bacterial DNA in terms of structure and evolution. And our previous study showed that mtDNA could induce the expression of STING to activate inflammation,^26^ we used immunoblot to detect the expression levels of STING after injection (Figure 2C). The results showed that the expression levels of STING were also parallel with the increases in neutrophil infiltration.

As neuroinflammation had been activated, we observed expressions of various inflammatory factors. Among them, the expression of IL‐1β, NLRP3 and IFN‐γ was upregulated (Figure 2D,E), and they were also consistent with the degrees of neutrophil infiltration. But, TNF‐α had no significant alteration after injection of fMLF, Aβ_1–42_, mtDNA and Aβ/mtDNA mixture compared to the control group, which indicated that in our experimental model, TNF‐α was not an effector in neutrophil infiltration. The expressions of inflammatory factors might explain the progressive aggravation of neuroinflammation in Alzheimer's disease.

### 3.3 Impaired neurons and their mitochondria, mtDNA induce neutrophil neuroinflammation

**FIGURE 3:**
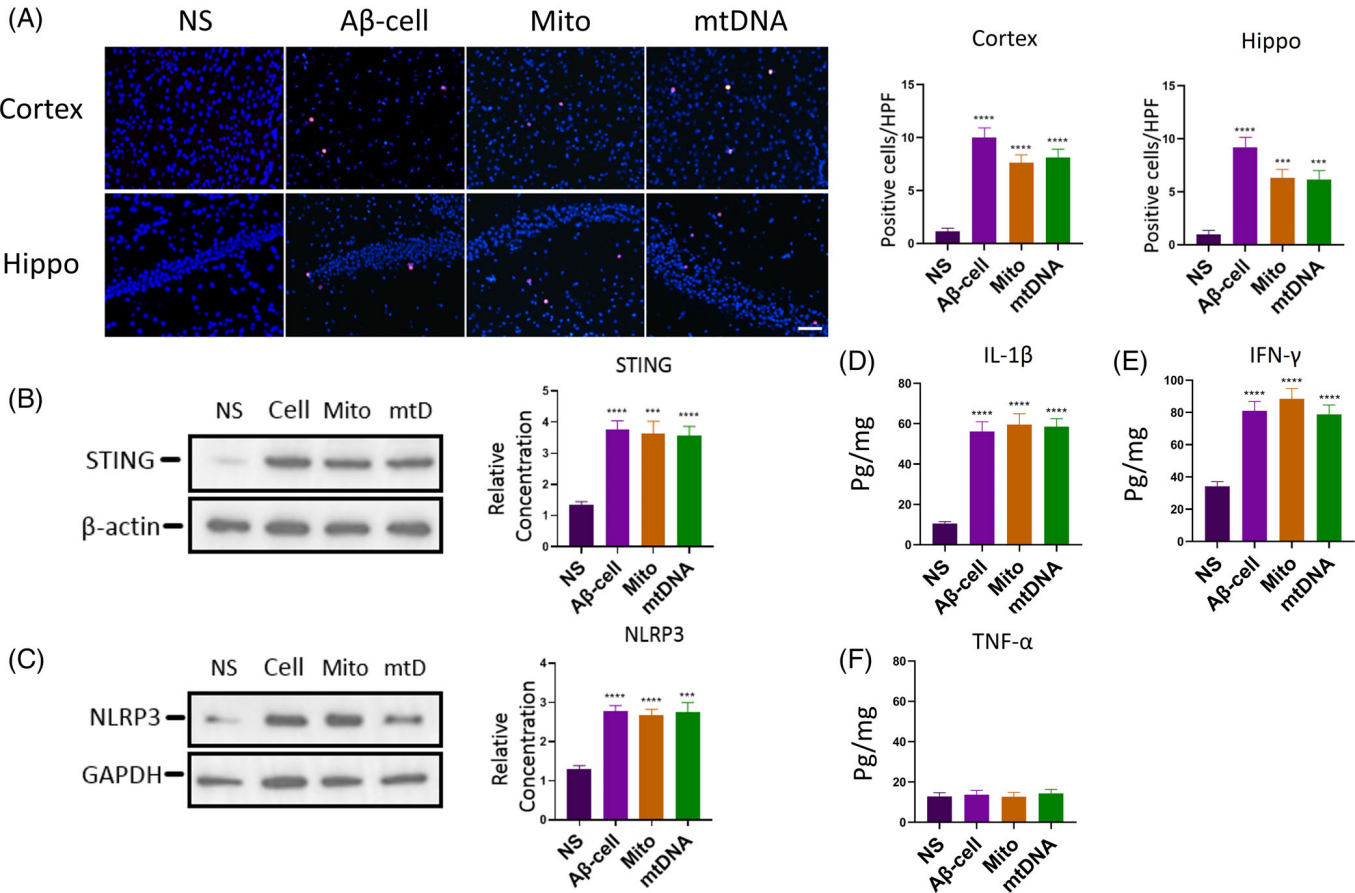
Neuroinflammation induced by Aβ_1–42_‐treated cells and their mitochondria, mtDNA. (A) HT22 cells were treated with Aβ_1–42_ to induce cellular apoptosis and necrosis, then their leaked mitochondria and mtDNA were isolated. These Aβ_1–42_‐treated cells, and their mitochondria, mtDNA were injected into mice brains, and tissues were collected after injection for 24 h. And Ly6G (red cells, DAPI blue) immunofluorescent stain was used to detect neutrophil infiltration, statistics. *n* = 6 per group. ***0.0001 < *p* < 0.001 versus NS; ****p < 0.0001 versus NS; *t* test; data are means ± SEM. Scale bar 75 μm. (B,C) Results of immunoblot with statistics of STING (B) and NLRP3 (C). *n* = 6 per group. ***0.0001 < *p* < 0.001 versus NS; ****p < 0.0001 versus NS; *t* test; data are means ± SEM. (D‐F) ELISA results of IL‐1β, IFN‐γ and TNF‐α expressions after injection for 24 h. *n* = 6 per group. ****p < 0.0001 versus NS; *t* test; data are means ± SEM.

Previously, we observed that Aβ_1–42_ could induce the apoptosis of neurons, a large number of mitochondria leakage and release mtDNA, which activated neutrophils infiltration. To verify that the initial chemotactic factors for neutrophils infiltration were Aβ damaged neurons with their intracellular constituents, we injected Aβ_1–42_‐treated hippocampus neurons HT22, as well as mitochondria and mtDNA leaked from these cells. We found that in the cerebral tissues of mice injected with Aβ_1–42_‐treated cells, mitochondria and mtDNA, neutrophil infiltrations were significantly increased (Figure 3A).

Besides, results showed that 24 h after injection, the expression of STING in mice cerebral tissues increased outstandingly (Figure 3B). In addition, for inflammatory factors, Aβ_1–42_‐treated cells and their mitochondria, mtDNA could also upregulate the expressions of NLRP3, IL‐1β and IFN‐γ (Figure 3C–E). Expression of TNF‐α had no significant change compared to the control group (Figure 3F). By injecting Aβ_1–42_‐treated hippocampus neurons, and their mitochondria and mtDNA, we found that these results were essentially the same as those of direct injections of Aβ_1–42_, untreated mitochondria and mtDNA. All of them could induce neutrophil infiltration and upregulate expressions of STING and inflammatory factors IL‐1β, NLRP3 and IFN‐γ. Namely, when neurons are exposed to Aβ_1–42_, their structure and metabolism could be damaged. Subsequently, a large amount of mitochondria and mtDNA were released. These cell constituents could chemotactically attract neutrophils infiltrated into cerebral tissues and upregulate the expressions of inflammatory factors, inducing neuroinflammation and disturbing physiological functions of the central nervous system.

### 3.4 Knocking out *STING* , *NLRP3* or *IL‐1β* gene inhibits neutrophil infiltration

Subsequently, we adopted specific gene knockout mice to detect the role of STING and inflammatory factor IL‐1, NLRP3 in neuroinflammation.

**FIGURE 4:**
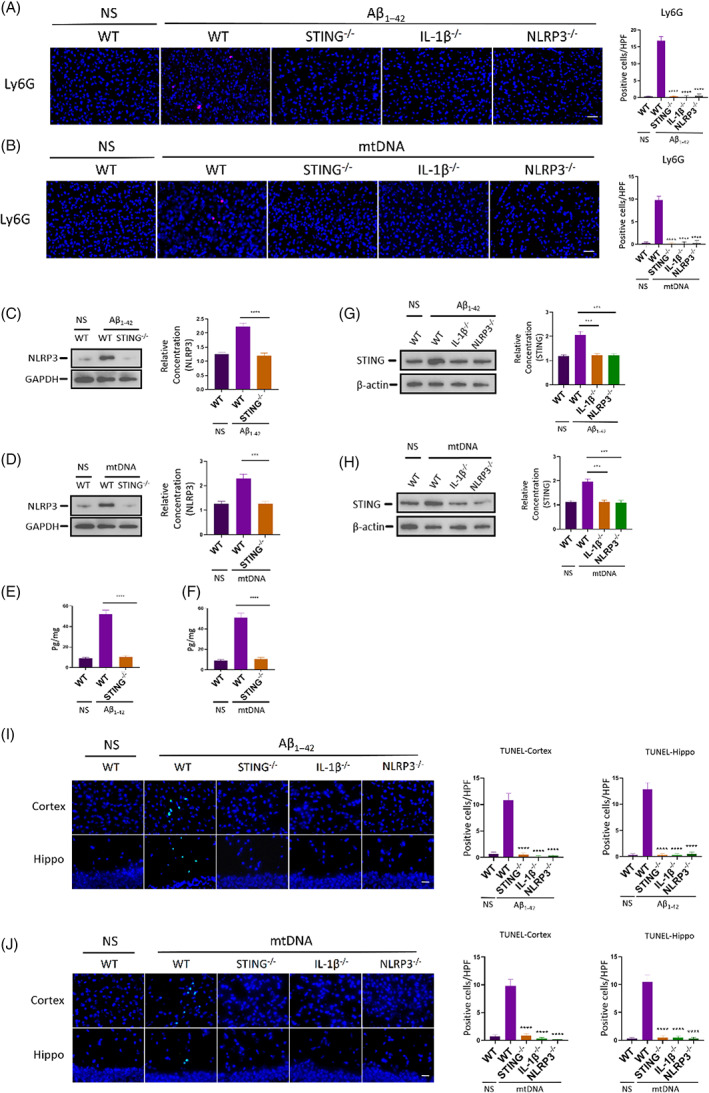
The inhibition of neutrophil infiltration and inflammatory cytokine expression in genetic knockout animals. (A,B) Aβ_1–42_ (A) and mtDNA (B) were injected into brain of *STING* (STING^−/−^), *IL‐1β* (IL‐1β^−/−^) and *NLRP3* (NLRP3^−/−^) gene knockout mice, respectively, 24 h after injection mice brain were harvested to detected neutrophil infiltration by immunofluorescent microscopy. *n* = 6. ****p < 0.0001 versus WT (Aβ_1–42_); ****p < 0.0001 versus WT (mtDNA); *t* test; data are means ± SEM. Scale bar 25 μm. (C‐F) NLRP3 (C,D) and IL‐1β (E,F) expressions of STING^−/−^ mice after 24 h injected Aβ_1–42_ and mtDNA. *n* = 6. ****p < 0.0001 versus WT (Aβ_1–42_); ***0.0001 < *p* < 0.001 versus WT (mtDNA); ****p < 0.0001 versus WT (mtDNA); *t* test; data are means ± SEM. (G,H) STING expression of IL‐1β^−/−^, NLRP3^−/−^ mice after 24 h injected Aβ_1–42_ (G) and mtDNA (H). *n* = 6. ***0.0001 < *p* < 0.001 versus WT (Aβ_1–42_); ***0.0001 < *p* < 0.001 versus WT (mtDNA); *t* test; data are means ± SEM. (I,J) The number of TUNEL+ cells in STING^−/−^, IL‐1β^−/−^, NLRP3 ^−/−^ mice brain after 24 h injection of Aβ_1–42_ (I) and mtDNA (J). *n* = 6. ****p < 0.0001 versus WT; *t* test; data are means ± SEM. Scale bar 25 μm.

First, we injected Aβ_1–42_ or mtDNA into the cerebra of *STING* (*STING* ^*−\−*^), *NLRP3* (*NLRP3* ^*−/−*^) and *IL‐1β* (*IL‐1β* ^*−\−*^) knockout mice. We observed that there were barely neutrophils in the brain (Figure 4A,B), and their neutrophil level was basically consistent with that of the control group. Additionally, the inflammatory factors NLRP3 (Figure 4C,D) and IL‐1β were also in trace levels (Figure 4E,F).

Second, we detected STING expressions in *NLRP3* ^*−/−*^ and *IL‐1β* ^*−\−*^ mice with an injection of Aβ_1–42_ or mtDNA, respectively. We found that STING was also downregulated on the condition of Aβ_1–42_ or mtDNA exposure (Figure 4G,H). These results demonstrated that NLRP3 and IL‐1β were also important factors in neutrophil infiltration. These interesting phenomena indicated that STING might be derived from neutrophils.

Additionally, from the results of the TUNEL apoptosis assay, we found that when knocked out one of *STING*, *NLRP3* or *IL‐1β* gene, not only neutrophil migration was inhibited, but also apoptotic cells were hardly to be found (Figure 4I,J). These results implied that silencing expressions of *STING*, *NLRP3* or *IL‐1β* gene could inhibit neutrophilic neuroinflammation and protect the central nervous system.

### 3.5 The relationship of mtDNA, *STING* and *NLRP3/IL‐1β*

**FIGURE 5:**
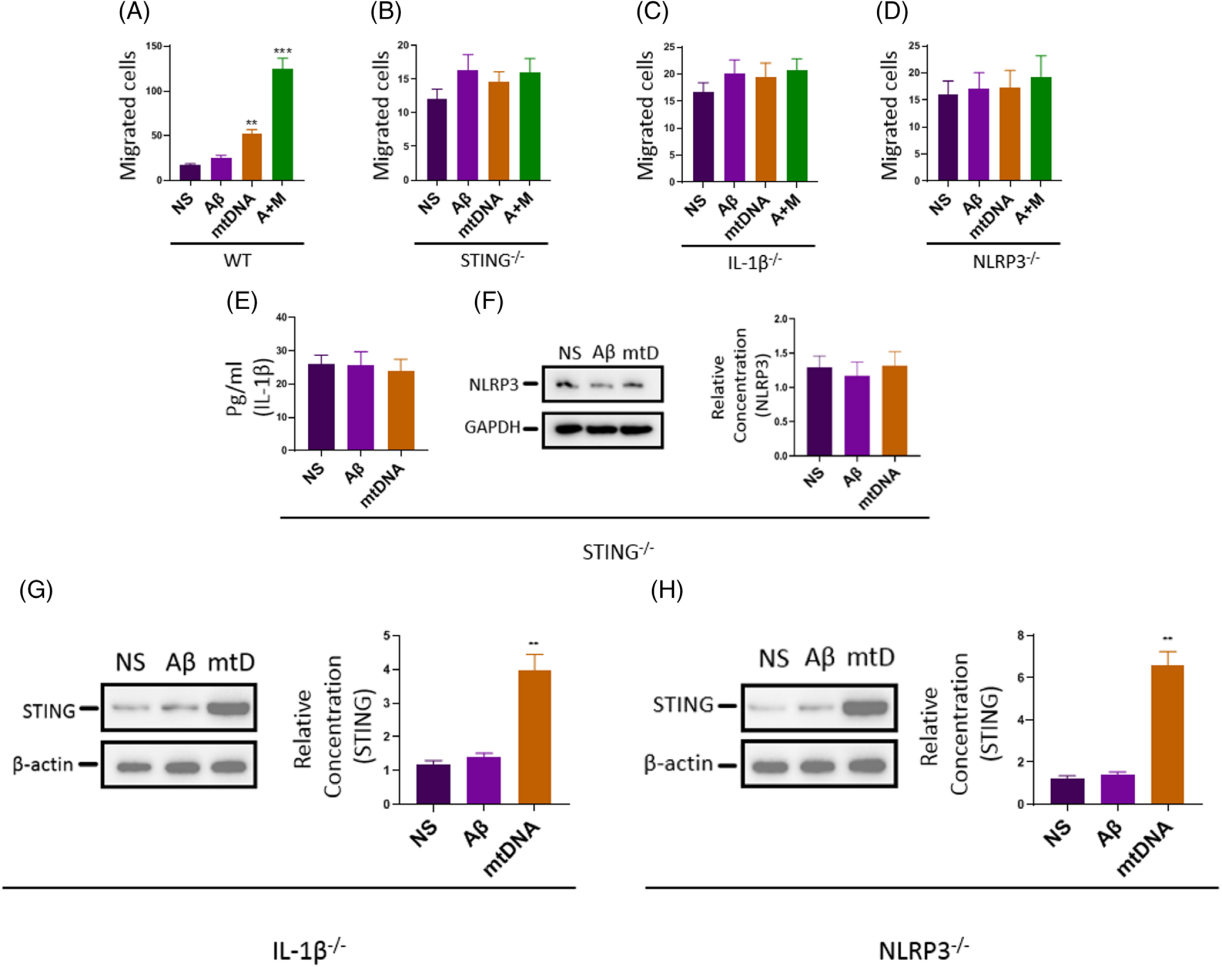
The exploration of neutrophil migration factors and the relationship between STING and NLRP3/IL‐1β. (A–D) The number of neutrophils extracted from Wild‐type (A), STING^−/−^(B), IL‐1β^−/−^(C), NLRP3 ^−/−^(D) mice migrated from the transwell inner chamber into outer chamber, stimulated by Aβ_1–42_, mtDNA and mixture (Aβ_1–42_/mtDNA, A+M) for 24 h. *n* = 3 per group. **0.001 < *p* < 0.01 versus NS; ***0.0001 < *p* < 0.001 versus NS; *t* test; data are means ± SEM. (E–F) ELISA results of IL‐1β expression (E) and immunoblot results of NLRP3 expression (F) of STING^−/−^ neutrophils, stimulated by Aβ_1–42_, mtDNA for 24 h. (G‐H) Immunoblot results of STING expression in IL‐1β^−/−^(G) and NLRP3 ^−/−^(H) neutrophils stimulated by Aβ_1–42_, mtDNA for 24 h. **0.001 < *p* < 0.01 versus NS; *t* test; data are means ± SEM.

To validate the relationship between neutrophil migration and these three genes, We extracted neutrophils from wild‐type, *STING* ^*−\−*^, *NLRP3* ^*−\−*^ and *IL‐1β* ^*−\−*^ mice and conducted transwell experiments. We observed that neutrophils of wild‐type mice could migrate towards higher concentrations of mtDNA. The Aβ/mtDNA mixture (A+M) had an enhanced chemotactic effect on neutrophils. But the number of migrated cells induced by Aβ_1–42_ did not have a significant difference to that of NS (Figure 5A). Besides, neutrophils extracted from *STING* ^*−\−*^, *NLRP3* ^*−\−*^ and *IL‐1β* ^*−\−*^ mice had little response with the stimulations of Aβ_1–42_ and mtDNA, even with the mixture (Figure 5B–D). In summary, *STING*, *NLRP3* and *IL‐1β* genes were crucial factors for neutrophil migration, when one of these genes is depleted, neutrophil migration would be inhibited.

Recently, researchers reported that STING might interact with NLRP3, and NLRP3 could hydrolyze pro‐IL‐1β into mature IL‐1β.^31^, ^32^, ^33^ So, we investigated their positional relationship in the signal transduction pathway. When neutrophils extracted from *STING* ^*−\−*^ stimulated by Aβ_1–42_ or mtDNA, no significant expression alterations were observed in IL‐1β and NLRP3 compared to the NS control group (Figure 5E,F). Nevertheless, when neutrophils from *NLRP3* ^*−\−*^ and *IL‐1β* ^*−\−*^ were exposed to mtDNA, the STING expression was upregulated (Figure 5G,H). But, it could not be increased by the stimulation of Aβ_1–42_. These results suggested that STING was the upstream factor of NLRP3 and IL‐1β. Meanwhile, mtDNA was the major initiator to STING instead of Aβ to induce the infiltration of neutrophils.

### 3.6 Neuron degeneration and cognitive impairment induced by Aβ_1–42_ and mtDNA are inhibited by knocking out *STING* , *NLRP3* or *IL‐1β* gene

**FIGURE 6:**
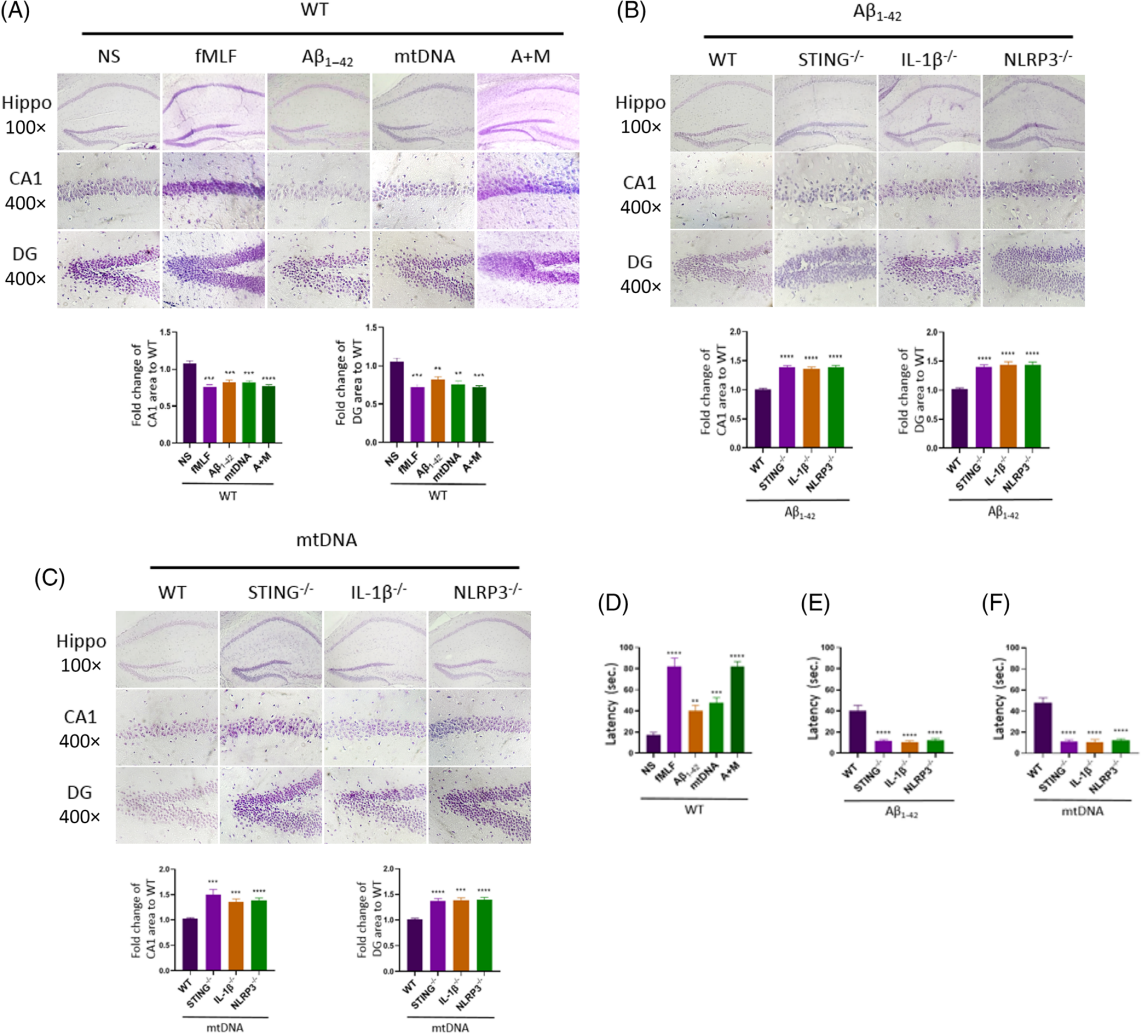
The alterations in cerebral tissues and cognitive impairment induced by Aβ_1–42_, mtDNA and mixture (A+M) in long‐term observation. (A) Images of hippocampus CA1 regions and DG (dentate gyrus) regions in wild‐type mice, stained by Nissl dye, with statistics of their area after injection for 5 weeks, *n* = 6 per group. **0.001 < *p* < 0.01 versus NS; ***0.0001 < *p* < 0.001 versus NS; ****p < 0.0001 versus NS; *t* test; data are means ± SEM. (B,C) Images and areas in CA1, DG regions of Aβ_1–42_ (B) and mtDNA (C) injected wild type, STING^−/−^, IL‐1β^−/−^, NLRP3 ^−/−^ mice, *n* = 6 per group. ***0.0001 < *p* < 0.001 versus WT; ****p < 0.0001 versus WT; *t* test; data are means ± SEM. (D‐F) Barnes Maze test for wild‐type (D) mice injected with Aβ_1–42_, mtDNA and Aβ_1–42_/mtDNA mixture (A+M) or STING^−/−^, IL‐1β^−/−^, NLRP3^−/−^ mice injected with Aβ_1–42_ (E) or mtDNA (F) for 5 weeks. *n* = 6 per group. **0.001 < *p* < 0.01 versus NS; ***0.0001 < *p* < 0.001 versus NS; ****p < 0.0001 versus NS; *t* test; data are means ± SEM.

In addition, we recorded the long‐term results after the injection of Aβ_1–42_ and mtDNA. For wild‐type mice, after 5 weeks of stereotactic injection, we took brain sections for experiments. We found that the hippocampus region atrophied, especially in CA1 and DG regions, which were sensitive to injury stimulation (Figure 6A). Compared with the control group, mice injected Aβ_1–42_ or mtDNA had significant atrophies in CA1 and DG regions. For the mice injected with Aβ/mtDNA mixture, their hippocampus had a severer atrophy to mice injected with Aβ_1–42_ or mtDNA. This confirmed that Aβ_1–42_ or mtDNA had toxicity to cerebral tissues, and their toxicity had a synergistic effect when they mixed together.

Next, we observed the cerebral changes in *STING* ^*−\−*^, *NLRP3* ^*−\−*^ or *IL‐1β* ^*−\−*^ knockout mice within 5 weeks period after injection. Results showed that hippocampus atrophies in CA1 and DG regions of genetic knockout animals were less severe than that of the control group when exposed to Aβ_1–42_ or mtDNA (Figure 6B,C). This indicated that the knockout of *STING*, *NLRP3* or *IL‐1β* gene could enhance the tolerance of cerebral tissue to impaired stimulations caused by Aβ_1–42_ or mtDNA.

Finally, we conducted Barnes maze cognitive test on mice. With 5 weeks exposed to Aβ_1–42_ or mtDNA, wild‐type mice had cognitive disorders manifested as the longer latency to find shelter (Figure 6D). When mice were injected with Aβ/mtDNA mixture, they had a severe cognitive disorders than that of Aβ_1–42_ or mtDNA‐injected mice. But when one of *STING*, *NLRP3* or *IL‐1β* gene knocked out, the cognition of mice had been improved (Figure 6E,F). These results were consistent with neutrophil migration degree and inflammatory factors expression levels. It proved that *STING*, *NLRP3* or *IL‐1β* gene had important roles in the progression of Alzheimer's disease. But as long as any knot on the mtDNA‐STING‐NLRP3/IL‐1β axis was knocked out, cerebra could enhance tolerance to injury stimulation caused by Aβ_1–42_ or mtDNA.

## 4 DISCUSSION

The neurotoxicity of Aβ_1–42_ has been widely reported before.^34^, ^35^, ^36^, ^37^ Our results confirmed the dose‐dependent toxicity of Aβ_1–42_, which was severer as the concentration increased. Although there were some controversies regarding the mechanism of the toxicity of amyloid protein, the neurotoxicity of length‐specific peptide Aβ_1–42_ has been verified by numerous researchers in their results.^14^, ^37^, ^38^, ^39^ Our data provided support on its neurotoxicity.

Additionally, recent reports showed that mitochondria had neuron‐impairment effect^40^, ^41^, ^42^ and mtDNA had an inflammatory‐promoting effect,^28^, ^29^, ^30^ but the pathological mechanism was still oblivious. Our results showed when mtDNA interacted with cerebral tissue, neutrophil infiltration was activated and the neuron apoptosis process was initiated. Results of in vitro experiments also revealed that mtDNA could induce neutrophil chemotactic migration. Neutrophils are harmful to neurons and our data are supplements and improvements for the mechanism of the mtDNA pathophysiology. Namely, when mtDNA is released from cells, STING protein which recognizes double‐stranded DNA will be activated. According to previous studies and our data on STING protein, it could bind to double‐stranded DNA from various sources, such as bacterial chromosome DNA, viral DNA and chromosome DNA fragments.^43^, ^44^, ^45^, ^46^, ^47^ This also explains the reason that mtDNA, with evolutionary homology to bacterial DNA, can induce neuroinflammation. The classic downstream product of STING is the molecules in the interferon family.^48^, ^49^ Our data showed that the expression of IFN‐γ was upregulated when STING was activated, which suggested that mtDNA could activate the STING pathway. In addition, recent studies found that STING interacted with NLRP3, and NLRP3 could validate pro‐IL‐1β into IL‐1β.^31^, ^32^, ^33^, ^50^, ^51^ Our results revealed that levels of NLRP, and IL‐1β increased parallelly, indicating that STING, NLRP3 and IL‐1β were in the same signal transduction pathway.

Furthermore, STING and NLRP3 expressions in neuro‐degeneration disease were reported by researchers.^16^, ^52^, ^53^ We found that STING, NLRP3 and IL‐1β participated in neutrophil neuroinflammation, but knocking out one of them could inhibit the injury progression. These results demonstrated that the activation of neutrophilic neuroinflammation requires mtDNA‐STING‐NLRP3/IL‐1β axis in integrity.

Surprisingly, we did not observe the upregulated expression of TNF‐α in both in vivo and in vitro experiments. This differs from previous research results.^22^, ^54^, ^55^ It implies that the level of TNF‐α is not dominated by Aβ_1–42_ or mtDNA. Therefore the role of TNF‐α in Alzheimer's disease models requires further exploration.

In conclusion, Alzheimer's disease is caused by neurodegeneration. In our research, we found that Aβ_1–42_ could induce neuron damage, following mitochondria and mtDNA leakage, which initiated cellular apoptosis and necrosis. The pathophysiological mechanism is that amyloid protein induces mtDNA presenting in the brain, which activates neutrophil migration into cerebra and aggravates neuron injury. The prerequisite of cerebral neutrophil infiltration is the integrity of the mtDNA‐STING‐NLRP3/IL‐1β axis. When any factor in this pathway is depleted, the migration of neutrophils into cerebral tissue will be ceased. Thus, the development of drugs or agents targeted on the knots of the mtDNA‐STING‐NLRP3/IL‐1β axis can inhibit neuroinflammation and alleviate the progression of Alzheimer's disease.

## AUTHOR CONTRIBUTIONS

Yuquan Wei and Xiawei Wei designed the study. Xiangyu Xia, Xuemei He and Tingmei Zhao managed the project, collected the data and prepared the manuscript. Xiangyu Xia, Jingyun Yang, Zhenfei Bi, Qianmei Fu, Jian Liu and Danyi Ao analysed the data.

## FUNDING INFORMATION

This work was supported by the National Science Foundation for Excellent Young Scholars (No. 32122052) and the National Natural Science Foundation Regional Innovation and Development (No. U19A2003).

## CONFLICT OF INTEREST STATEMENT

All authors declare no competing interests.

## Data Availability

All data included in this study are available upon request by contact with the authors.
